# Biological Potential and Bioaccessibility of Encapsulated Curcumin into Cetyltrimethylammonium Bromide Modified Cellulose Nanocrystals

**DOI:** 10.3390/ph16121737

**Published:** 2023-12-17

**Authors:** Francisca Casanova, Carla F. Pereira, Alessandra B. Ribeiro, Pedro M. Castro, Ricardo Freixo, Eva Martins, Diana Tavares-Valente, João C. Fernandes, Manuela E. Pintado, Óscar L. Ramos

**Affiliations:** CBQF—Centro de Biotecnologia e Química Fina-Laboratório Associado, Escola Superior de Biotecnologia, Universidade Católica Portuguesa, Rua Diogo Botelho 1327, 4169-005 Porto, Portugal

**Keywords:** curcumin, delivery system, CNC-CTAB, biological potential, intestinal permeability

## Abstract

Curcumin is a natural phenolic compound with important biological functions. Despite its demonstrated efficacy in vitro, curcumin biological activities in vivo are dependent on its bioaccessibility and bioavailability, which have been highlighted as a crucial challenge. Cetyltrimethylammonium bromide-modified cellulose nanocrystals (CNC-CTAB) have been shown to be effective in curcumin encapsulation, as they have the potential to enhance biological outcomes. This study evaluated the biological effects of curcumin encapsulated within CNC-CTAB structures, namely its antioxidant, anti-inflammatory and antimicrobial properties, as well as the release profile under digestion conditions and intestinal permeability. Encapsulated curcumin demonstrated antioxidant and anti-inflammatory properties, effectively reducing reactive oxygen species and cytokine production by intestinal cells. The delivery system exhibited antimicrobial properties against *Campylobacter jejuni* bacteria, further suggesting its potential in mitigating intestinal inflammation. The system showed the ability to protect curcumin from degradation and facilitate its interaction with the intestinal epithelium, highlighting the potential of CNC-CTAB as carrier to enhance curcumin intestinal biological functions.

## 1. Introduction

Curcumin is a natural lipophilic phenolic compound derived from the rhizome of turmeric (*Curcuma longa*) [1]. Various in vitro and pre-clinical cell culture studies have demonstrated that curcumin possesses extensive biological activity as an antioxidant, antimicrobial, anti-inflammatory, anti-diabetic, anti-cancer and wound-healing agent [2,3,4]. Different studies also suggest a potential therapeutic role for curcumin in numerous chronic diseases, such as colon cancer and inflammatory bowel diseases [3]. Curcumin has a diverse range of molecular targets like transcription factors, growth factors (and their receptors), cytokines, enzymes and genes regulating cell proliferation and apoptosis, all of which have been reviewed by Goel et al. [5]. Clinical studies dealing with the efficacy of this compound in humans can also be cited [6,7,8,9]. In spite of its demonstrated efficacy, the limited bioavailability of curcumin has been highlighted as a major problem. Studies over the past three decades related to absorption, distribution, metabolism and excretion have revealed poor absorption and rapid metabolism, which severely curtail its bioavailability. Studies revealing curcumin’s low serum levels, limited tissue distribution, apparent rapid metabolism and short half-life have been reviewed [10,11]. Because of the poor bioavailability, very high oral doses and repeated dosing have been used to obtain effective plasma levels, with mixed results, as high doses of curcumin may cause gastric disturbance, often resulting in poor patient compliance [8,9].

These limitations can potentially be overcome by using enabling delivery systems, which may be able to preserve and enhance the functionality of active biomolecules, by improving its bioaccessibility and bioavailability and allowing for a sustained release [12]. Interest in cellulose materials as carriers for lipophilic molecules has increased recently due to their unique physicochemical properties, as reviewed by Casanova et al. [12]. Nevertheless, surface modification or coupling with other polymers is often employed to modulate and optimize the loading and release of biomolecules [12]. Aminated cellulose nanocrystals (CNC) [13]; oxidized-CNC [14]; hydrophobic modified CNC [15,16]; and CNC coupled with chitosan [17,18], cyclodextrins [19] and poly(lactic-co-glycolic) acid [20] have been employed to encapsulate curcumin. In our previous work, cetyltrimethylammonium bromide (CTAB)-modified CNC proved to be a promising curcumin delivery system, with a high encapsulating efficiency and sustained release properties [21]. Furthermore, curcumin encapsulation into this system allowed the reduction of its cytotoxicity by allowing a 6-fold dose increase, highlighting the potential of this system to deliver curcumin. This system has also been explored in the literature for the encapsulation of curcumin, showing high encapsulation efficiencies over 80% [15]. However, the authors only explored structural and encapsulation properties. The influence of such encapsulations on the bioactivities of curcumin has not been assessed or addressed before, but is of utmost importance to understand the impact of the delivery system on biological functions and applicability. 

In this work, the biological potential of encapsulated curcumin into CTAB-modified CNC, as well as free curcumin and CNC-CTAB structures, was assessed through in vitro experiments. Antioxidant, anti-inflammatory and antimicrobial activities were explored. Additionally, an in vitro model simulating gastrointestinal (GI) digestion was utilized to assess the impact of the digestion conditions on the release profile of curcumin. After digestion, the intestinal permeability of the bioaccessible molecule was evaluated using an in vitro co-culture model of Caco-2/HT29-MTX intestinal cells. To the best of our knowledge, this is the first study investigating the in vitro antioxidant, immunomodulatory and antimicrobial effects of CNC-CTAB delivery systems, particularly those encapsulating curcumin. This study is unique as it provides comprehensive evaluations of biological potential, release profile and intestinal permeability of CNC-CTAB-encapsulated curcumin, which is of vital importance to assess the applicability of the system. 

## 2. Results and Discussion

### 2.1. Characterization of Encapsulated Curcumin Particles

**Figure 1:**
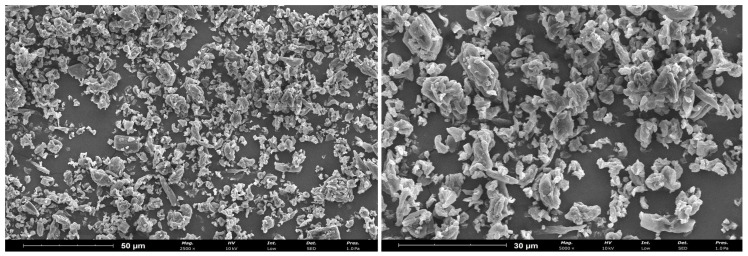
SEM images of spray-dried CNC-CTAB encapsulating curcumin (2500×/5000×, 10 kV, 1.0 Pa, scale bars equal 50 µm and 30 µm).

Curcumin was encapsulated into CTAB-modified CNC followed by spray-drying, one of the most commonly used drying methods in industrial applications, due to its flexibility, easy scale-up, control of structure properties and relative low process cost [22]. The obtained encapsulated curcumin particles were characterized in terms of yield, encapsulation efficiency (EE), loading capacity (LC), zeta potential (ZP), size and morphology. Results are presented in Table 1 and Figure 1.

CNC-CTAB delivery system encapsulating curcumin showed a promising EE of ca. 80%, corroborating the results previously obtained in the literature [15,21]. The binding is probably due to the interaction between curcumin’s benzene rings with the hydrophobic region of CNC-CTAB (C-H moieties) via electrostatic and hydrophobic interactions, which, in combination with hydrogen bonding, is effective in curcumin encapsulation [21]. Furthermore, the adsorbed surfactant monomers can reorganize and induce hydrophobic interactions between the alkyl chains of the surfactant to form surfactant clusters, further favoring curcumin binding [15,21]. The system was produced with a loading capacity of ca. 28% and a mass yield of ca. 82%, which is a promising result for laboratory scale spray-drying processes, as these are reported to be in the range of 20–70% [23]. CNC-CTAB encapsulating curcumin is moderately stable, with a negative ZP value of ca. −18 mV. Results from particle size analysis performed via laser diffraction showed a D(4:3) mean diameter of 9.34 µm, with 50% of the sample volume containing particles below 7.33 µm (Dv(50)). These results show that even though a nanomaterial was used to construct the delivery system, the spray-drying process has mostly increased the particle size from nano- to microscale. This is particularly interesting from a commercial perspective, as materials at the microscale may reach the market more easily due to regulatory restrictions associated with the use of nanomaterials. SEM images (Figure 1) showed irregular, mostly spheroid particles with diameters of 2–10 µm, corroborating particle size analysis results. Most of the microstructures displayed wrinkled surfaces with depressions formed on the surface of the structures, which may be due to shrinkage during drying and cooling with the consequent deformation of its surface [24]. Very similar morphologies have been obtained in the literature for spray-dried nanocellulose delivery systems [24,25]. Certain amounts of curcumin seem to be present as free crystals (smoother, not spherical particles) that dried separately. This is possibly a consequence of precipitation that occurred before the spraying process, when the biomolecule solution was added to the CNC suspension, or it may be a consequence of different drying kinetics of biomolecules and CNC [25].

### 2.2. Biological Potential

Curcumin has been described to possess antioxidant, anti-inflammatory and antibacterial capabilities [26,27]; however, the influence of encapsulation on these bioactivities must be assessed in order to evaluate the applicability of the delivery system. Thus, the biocompatible concentrations of each sample determined in our previous work, namely 0.12 g/L for encapsulated curcumin, 0.005 g/L (15 µM) for free curcumin and 0.09 g/L for free CNC-CTAB [21], were the concentrations used for biological activity assessment.

#### 2.2.1. Antioxidant Capacity

**Figure 2:**
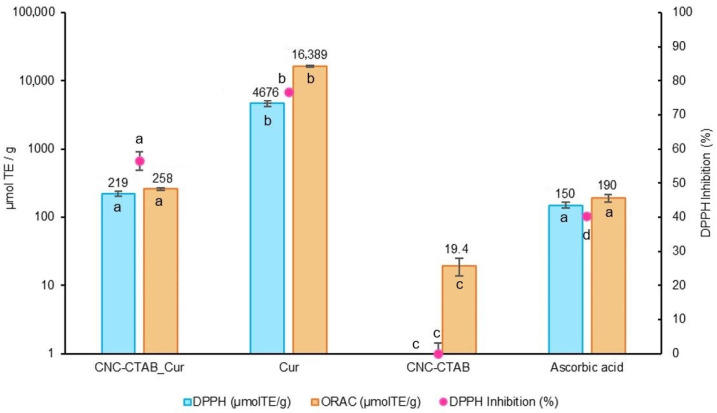
Antioxidant activity of CNC-CTAB encapsulating curcumin, alongside free curcumin and carrier (CNC-CTAB) in comparison to ascorbic acid (used as positive control) according to the DPPH and ORAC assays. Different letters represent the statistically significant (*p* < 0.05) differences found between the different samples for each assay performed.

Firstly, the samples were assayed for their intrinsic scavenging/reducing activity through radical scavenging assays, namely the DPPH free radical scavenging assay and the oxygen radical absorbance capacity assay (ORAC). Ascorbic acid (vitamin C) was used as the antioxidant control. Results are presented in Figure 2.

From Figure 2 it is possible to observe that curcumin exhibited powerful antioxidant activity in both, DPPH (4675.64 µmol TE/g, 76.69% DPPH inhibition) and ORAC (16,388.63 µmol TE/g) assays, which is consistent with previous studies that attribute a similar effect to the presence of phenolic groups in the biomolecule [3]. The differences in the Trolox equivalent antioxidant capacity between the two methods may arise from the different mechanisms involved in each methodology [28]. Similar antioxidant results for curcumin of 72.07% DPPH scavenging activity and 14,981.34 µmol TE/g ORAC value have been reported in the literature [29]. Curcumin antioxidant activity decreased (*p* < 0.05) after encapsulation, as indicated by lower DPPH scavenging activity (219.39 µmol TE/g, 56.50% DPPH inhibition) and ORAC value (257.79 µmol TE/g). This decrease can be attributed to the reduced availability of free phenolic groups in the encapsulated curcumin. However, the CNC-CTAB delivery system encapsulating curcumin still showed an antioxidant activity similar to that of ascorbic acid (*p* > 0.05), demonstrating a promising antioxidant potential. Previous studies have also reported an initial decrease in curcumin’s antioxidant capacity after encapsulation, followed by an increase over time, suggesting that encapsulation can protect curcumin from degradation and preserve its antioxidant activity [30].

##### Production of Reactive Oxygen Species (ROS)

**Figure 3:**
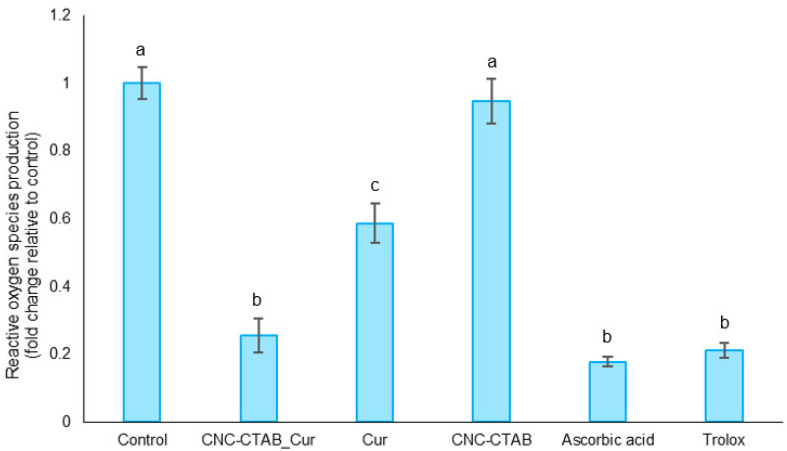
Antioxidant potential of CNC-CTAB encapsulating curcumin, free curcumin and CNC-CTAB, ascorbic acid and Trolox, by measuring its impact on the production of ROS by Caco-2 cells in the presence of an oxidizing agent (H_2_O_2_), relative to the basal level (control). Different letters represent the statistically significant (*p* < 0.05) differences found between the samples.

Although radical scavenging methods provide information about the chemical antioxidant activity of samples, it is important to consider that the biological antioxidant potential can be influenced by factors which cannot be predicted solely from these values [31]. Hence, we examined the antioxidant ability of CNC-CTAB encapsulating curcumin, as well as the free biomolecule and carrier at the highest non-cytotoxic concentrations, into counteracting H_2_O_2_-induced oxidative stress in intestinal Caco-2 cells [21], by evaluating the production of reactive oxygen species (ROS) by these cells. Results are presented in Figure 3.

Curcumin, at the highest non-cytotoxic concentration (15 µM), was able to reduce (*p* < 0.05) the production of ROS by Caco-2 cells when subjected to an oxidizing agent (H_2_O_2_) by ca. 40%. Investigations by different groups have confirmed that during free radical reactions, the most easily abstractable hydrogen from curcumin is from the phenol-OH group, resulting in the formation of phenoxyl radicals. These phenoxyl radicals are stabilized across the keto-enol structure and are less reactive than peroxyl radicals, providing protection against ROS-induced oxidative stress [32]. The regeneration reaction of phenoxyl radicals back to curcumin by water soluble antioxidants like ascorbic acid, impart the molecule with a chain breaking antioxidant ability like vitamin E [33]. Curcumin was also described to induce endogenous antioxidant defense mechanisms through gene regulatory mechanisms. It activates the redox-regulated transcription factor Nrf2 (nuclear factor (erythroid-derived 2)-like 2), which in turn induces the expression of antioxidant enzymes such as paraoxonase 1 (PON1), heme oxygenase 1 (HO1) and glutathione [26,34,35]. The ROS levels in cells treated with CNC-CTAB were comparable to the basal level (*p* > 0.05), indicating that this material does not possess inherent antioxidant capacity. Encapsulated curcumin was able to significantly reduce (*p* < 0.05) the production of ROS by Caco-2 cells subjected to the oxidizing agent by approximately 75%. This capacity can be compared to that of positive control molecules, Trolox and ascorbic acid (*p* > 0.05). Encapsulated curcumin exhibited greater reduction in ROS production compared to the free biomolecule at its safe concentration towards intestinal cells. This enhancement in antioxidant capacity can be attributed to the higher concentration of curcumin in the encapsulated system, which is released from the carrier over time. By maintaining safe levels towards cells, the encapsulated system allowed for improved antioxidant activity. Sampath et al. [36] have also reported that curcumin encapsulation resulted in prolonged circulation and increased interaction between curcumin and freed radicals, thereby potentiating its antioxidant capacity [36]. Although the antioxidant activity observed by radical scavenging methods decreased after encapsulation, the opposite trend was observed when the production of ROS by cells was analyzed, as this assay extends over a longer period (24 h), allowing for the gradual release of the biomolecule from the carrier and favoring sustained antioxidant activity while maintaining safe biomolecule levels.

#### 2.2.2. Immunomodulation

**Figure 4:**
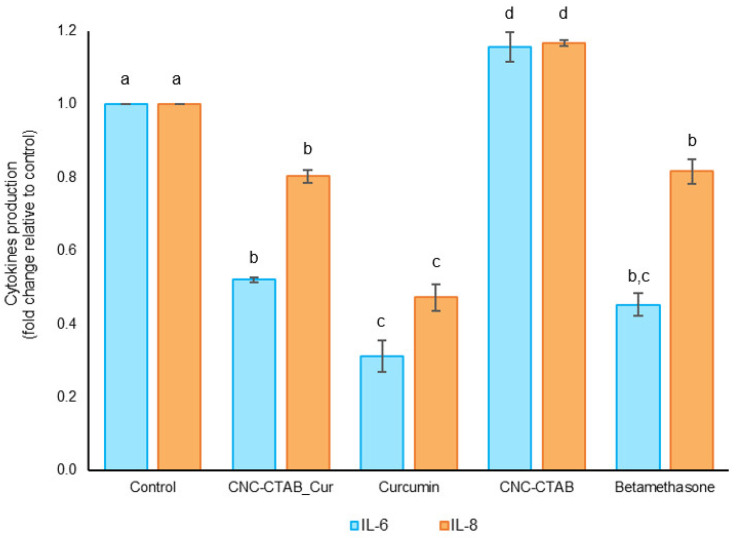
Immunomodulatory effect of CNC-CTAB encapsulating curcumin, free curcumin and carrier, and betamethasone on Caco-2 cell line using IL-1β as a pro-inflammatory stimulus in relation to the basal level (control). Different letters represent the statistically significant (*p* < 0.05) differences found between the samples for each cytokine.

The present study evaluated the effect of CNC-CTAB delivery systems encapsulating curcumin, as well as free curcumin and carrier, on the inflammatory response of Caco-2 cells stimulated by IL-1β, an important intestinal inflammatory mediator [37,38,39]. The production of cytokines IL-6 and IL-8 was measured, and betamethasone used as an anti-inflammatory control. The results are presented in Figure 4.

In cells stimulated with IL-1β, curcumin showed a relevant anti-inflammatory effect as demonstrated by the significant reduction in the secretion of selected cytokines. Curcumin led to a reduction in IL-6 and IL-8 (*p* < 0.05) by approximately 70% and 50%, respectively, compared to the stimulated control cells, indicating a positive influence upon the inflammatory response of Caco-2 cells (Figure 4). The anti-inflammatory effects of curcumin have been reported in numerous in vitro and in vivo studies [26]. These effects have been reported to be related to the inhibition of the transcription factor NF-κB by curcumin, as evidenced by its ability to inhibit NFκB–DNA interaction as well as the expression of NF-κB target genes [40,41,42,43,44]. The anti-inflammatory effect of curcumin decreased after encapsulation, reducing the secretion of IL-6 and IL-8 by ca. 50% and 20%, respectively (*p* < 0.05). This decrease in anti-inflammatory response may be related to the slight pro-inflammatory effect exerted by the CNC-CTAB carrier in its free form, which led to an increase in IL-6 and IL-8 production by ca. 20% (*p* < 0.05). Nevertheless, encapsulated curcumin was able to reduce cytokine production to levels similar to the anti-inflammatory agent betamethasone, indicating the potential anti-inflammatory activity of this system. Previous studies have also reported anti-inflammatory effects for curcumin-encapsulated systems. For example, in RAW 264.7 cells, curcumin-loaded polymeric nanostructures diminished the production of pro-inflammatory cytokines-IL-1β, IL-6 and TNF-α in macrophages stimulated via Aβ fibers and LPS [45]. Curcumin encapsulated in nanostructures has also been found to decrease the expression of TNF-α and IL-8 in THP-1 cells, and reduce NO production to levels close to unstimulated cells [46,47].

#### 2.2.3. Antimicrobial Activity

The antimicrobial activity of CNC-CTAB encapsulating curcumin, as well as the free biomolecule and the free carrier material, was evaluated against common pathogenic microorganisms associated with GI disorders. The previously determined biocompatible concentrations [21] were tested through the incorporation method due to the aqueous insolubility of the delivery system. Four reference strains were selected: *Escherichia coli*, *Salmonella enteritidis*, *Listeria innocua* and *Campylobacter jejuni*, representing both Gram-positive and Gram-negative bacteria. Results are presented in Table 2.

Table 2 shows that none of the samples at the tested concentrations were able to inhibit the growth of *Escherichia coli*, *Salmonella enteritidis* and *Listeria innocua* using the employed method. The reported minimum inhibitory concentration (MIC) values of curcumin against these strains are higher than the concentrations tested in this study, which may explain the lack of antimicrobial activity observed [48,49]. Curcumin has been reported to have a MIC of approximately 430 µM for *E. coli* and *S. enteritidis*, and 340 µM for *Listeria monocytogenes* [50,51,52]. However, when observing the effects upon *Campylobacter jejuni*, the results are quite different. Free curcumin at the tested concentration had no impact on the growth of the microorganism, but free CNC-CTAB and CNC-CTAB encapsulating curcumin were able to inhibit its growth. Curcumin exerts antimicrobial actions through various mechanisms, including the inhibition of DNA replication, the modification of plasmid gene expression, cell membrane deterioration and motility reduction [49]. Nevertheless, despite the high curcumin concentration present in the delivery system, its lipophilic nature makes its release within the agar media difficult, and so the antimicrobial results obtained in this study for this system are probably due to CNC-CTAB, as corroborated by the antimicrobial effect exerted by the free carrier material. Although CNC has not been reported to exert relevant antimicrobial activity [53], CTAB surfactant has shown good antimicrobial properties attributed to its positive charge in an amphipathic structure [54]. In fact, a study by Som No et al. [55] evaluated the antibacterial properties of curcumin nanoparticles formulated with surface-charged surfactants against *L. monocytogenes*, which contains a highly negatively charged lipid bilayer [55]. The CTAB formulation (positive charge) showed the highest inhibitory effect, with MIC values (1 µg/mL) significantly lower than the ones obtained for unprocessed curcumin (350 µg/mL). The antimicrobial properties observed for CNC-CTAB encapsulating curcumin against *C. jejuni* are very promising considering a gastrointestinal delivery of the system within nutraceutical applications, as *Campylobacter jejuni* is one of the most prevalent causes of bacterial enteritis and colon inflammation [56]. 

### 2.3. Release Profile under Digestion Conditions

**Figure 5:**
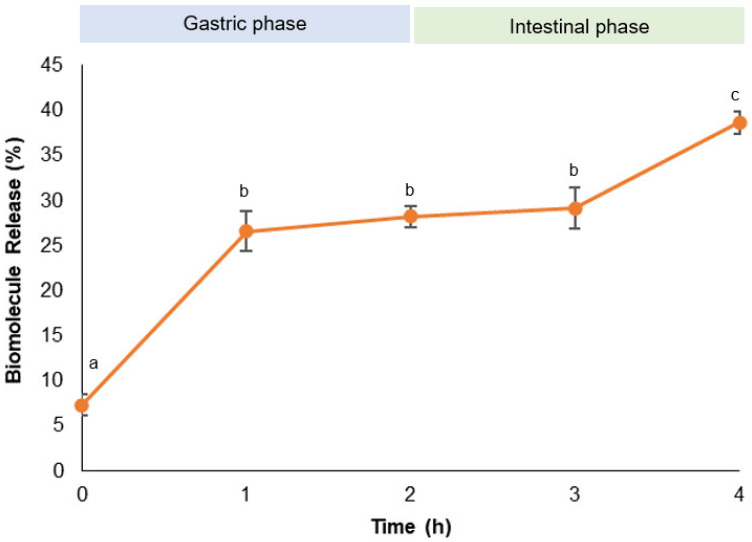
Release of curcumin throughout the in vitro gastrointestinal digestion from CNC-CTAB delivery system. Different letters represent statistically significant (*p* < 0.05) differences found between time points for each biomolecule.

An in vitro simulation of the human gastrointestinal system was used to mimic the physiological conditions of digestion and evaluate the release profile of curcumin from CNC-CTAB throughout the GI tract. The standardized European protocol developed by INFOGEST for the static in vitro simulation of GI food digestion [57,58] was applied to the delivery system and free curcumin. The release results are presented in Figure 5.

Curcumin release profile from the CNC-CTAB delivery system under simulated digestion conditions exhibited an initial burst release within the first hour, with approximately 25–30% of curcumin released. This was followed by a slower release phase, where up to 40% of curcumin was released over 4 h (Figure 5). The initial burst release may be due to the acidic conditions of the gastric phase, which can weaken the hydrogen bonds between cellulose and curcumin, facilitating its release. Furthermore, the increased positive charge of CTAB at acidic pH values can induce electrostatic repulsions between CTAB molecules, leading to the disruption of surfactant clusters that entrapped curcumin. Curcumin unbinding and faster release in acidic conditions have also been observed by Iurciuc-Tincu et al. [59]. Typical release profiles from cellulose nanostructures characterized by a biphasic trend with a fast initial burst release in the first few hours (0.5–2 h) followed by a slower release phase have been reported in the literature [12].

### 2.4. Intestinal Permeability

**Figure 6:**
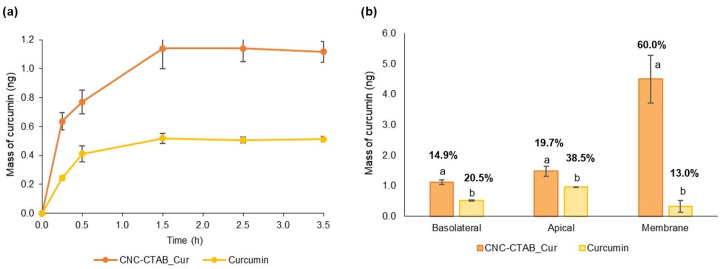
(**a**) Cumulative intestinal permeability of free and encapsulated curcumin across Caco-2 and HT29-MTX co-culture over 3.5 h; (**b**) amount of curcumin present in the basolateral side, apical side and within the intestinal epithelium membrane, both in absolute mass (ng) and in percentage in relation to the initial curcumin (percentage values shown in the graph) after 3.5 h of exposure. Different letters represent the statistically significant (*p* < 0.05) differences found between the samples for each compartment.

Aqueous solubility and intestinal permeability play a critical role in oral bioavailability, and thus the biological activity of a compound [60]. Curcumin has been shown to exhibit poor absorption and bioavailability due to its poor aqueous solubility and instability/degradability [61,62,63,64]. The intestinal permeability of curcumin after digestion was evaluated through an in vitro co-culture model of Caco-2/HT29-MTX cells. This model simulates the passage of compounds from the apical (i.e., luminal) to the basal (i.e., blood) side of the GI tract, simulating the passive diffusion and transport through enterocytes of the small intestine [65]. The cytotoxic effects of the digested samples were determined against Caco-2 and HT29-MTX cells, and a 1:10 dilution after digestion presented no cytotoxic effects towards both cell lines (Supplementary Information, Figure S2). The digested CNC-CTAB encapsulating curcumin, as well as the digested free curcumin and carrier material, were added to the apical compartment of the Caco-2/HT29-MTX co-culture model, and the permeability of the released curcumin to the basolateral side was measured over a 3.5 h period. At the end of the experiment, the quantity of curcumin present at the apical side and entrapped within the membrane (after cell disruption) was also assessed. Figure 6a illustrates the cumulative intestinal permeability of curcumin over a 3.5 h period, while Figure 6b displays the distribution of curcumin in the basolateral side, apical side and within the intestinal epithelium membrane, both in absolute mass (ng) and as a percentage relative to the initial added curcumin (percentage values shown in the graph).

The permeation study revealed that both curcumin and encapsulated curcumin exhibited an initial rapid permeation within the first 30 min, followed by a slower permeation phase occurring until 1.5 h, after which no further biomolecule permeation was observed. Similar curcumin permeation profiles have been observed in the literature [66]. The higher amount of curcumin that permeated from the delivery system can be attributed to the higher initial concentration of curcumin in this sample, made possible by the reduction in cytotoxicity achieved through encapsulation [21]. Interestingly, the percentage of added curcumin that was able to permeate through the intestinal epithelium was slightly higher for free curcumin (ca. 20%) than for encapsulated curcumin (ca. 15%). Additionally, free curcumin presented a higher apparent permeability value (3.43 ± 0.09 × 10^6^ cm/s) compared to encapsulated curcumin (2.51 ± 0.13 × 10^6^ cm/s) (*p* < 0.05). The adhesion of encapsulated particles to the mucus produced by HT29-MTX cells, which mainly consists of negatively charged glycoproteins, may contribute to the slower permeation of encapsulated curcumin [67]. Various studies indicate that the mucus layer, in addition to affording protection, also poses a potential barrier to compound absorption [68]. The permeability of positively charged moieties, which is the case of CTAB, may be slowed down by binding to the mucus layer. Moreover, mucus selectivity and permeability are sensitive to the scale properties of the particles, and the micro-size of the spray-dried particles together with the surfactant clusters formed may be also at play here [69]. Guri et al. [66] also found that encapsulated curcumin was retained significantly more in the mucus layer compared to control samples, with an impact on curcumin absorption rate [66]. On the other hand, in a study by Akbari et al. [70], the cellular uptake of encapsulated coumarin was found to be a saturable process, pointing to the involvement of receptor-mediated endocytosis. The authors also suggested that the involved receptors may interact more efficiently with negatively-charged particles [70]. Nevertheless, the encapsulation of curcumin, by reducing curcumin cytotoxic levels, allows for the delivery of higher amounts of curcumin through the intestinal epithelium, potentiating its biological functions throughout the body. Furthermore, curcumin encapsulation was able to reduce the percentage of curcumin present at the apical side of the intestinal epithelium and significantly increase the amount of curcumin entrapped within the epithelium membrane, likely due to its mucoadhesive properties and immobilization in the luminal mucus layer [70]. From a delivery perspective, mucus acts as an additional barrier limiting diffusion but also potentially increases the residence time of mucoadhesive particles near the epithelium. This allows curcumin to interact with the intestinal epithelium cells, namely Caco-2 cells, towards which it has shown interesting biological activities throughout our work. The amount of curcumin delivery system remaining in the gut is also beneficial for exerting antimicrobial activity against gastrointestinal pathogens such as *C. jejuni.* The fact that quantified curcumin in the three compartments (apical, basolateral and membrane) only accounts for 70% of the initial free curcumin, while encapsulated curcumin accounts for 95%, demonstrates the protective effect of the delivery systems against degradation. Detailed mass balance studies in the literature have also shown the loss of free curcumin during transport, which has been attributed to its degradation in aqueous solution at neutral and alkaline pH [61,66]. Particularly, curcumin instability and degradation in HBSS (Hanks’ Balanced Salt Solution) permeation buffer has been reported [63]. The capacity of the delivery system to protect curcumin from gastrointestinal degradation may be related to the anti-digestive properties of cellulose structures, which contribute to the enhancement of curcumin bioaccessibility [64].

## 3. Materials and Methods

### 3.1. Reagents and Materials

The reagents used in the experiments were of analytical grade or higher. Commercial CNC with a needle-like morphology with 10–20 nm width and 50–400 nm length was kindly supplied by the Cellulose Lab (Fredericton, NB, Canada). CTAB (cetyltrimethylammonium bromide) (>99.0%) was purchased from Sigma-Aldrich (St. Louis, MO, USA). CNC modification with CTAB was performed as described in Casanova et al. [21]. Curcumin was purchased from Alfa Aeser (Haverhill, MA, USA). Trolox ((±)-6-hydroxy-2,5,7,8-tetramethylchromane-2-carboxylic acid) and ascorbic acid (>99%) were purchased from Sigma-Aldrich.

### 3.2. Curcumin Encapsulation and Spray Drying

Calculated amounts of curcumin were dissolved in ethanol, and a CNC-CTAB aqueous suspension was added to the ethanolic solution in order to achieve a final CNC-CTAB concentration of 2% (*w*/*v*) and bioactive:carrier ratio of 1:3 in ethanol (70%, *v*/*v*). The mixture was stirred at room temperature for 30 min. The bioactive-loaded particles were collected through centrifugation at 10,000 rpm for 10 min at room temperature. The unbound curcumin that remained in the ethanolic supernatant was quantified and the pellet was washed with distilled water via centrifugation twice. Drying was performed using a BÜCHI B-290 Mini Spray Dryer (Flawil, Switzerland) with a standard 0.5 mm nozzle. The mixture (0.75%, *w*/*v*) was fed into the spray dryer at a flow rate of 4 mL/min (15%) and an inlet temperature of 120 °C. Spray gas flow, aspiration rate and nozzle cleaner were set to 670 L/h, 75% (30 m^3^/h) and 0, respectively.

### 3.3. Characterization of Encapsulated Curcumin Particles

The encapsulation yield (%) was expressed as the ratio of the final mass obtained and the mass of the starting materials, according to Equation (1). The unbound curcumin (*Cur_unbound_*) that remained in the supernatant after curcumin loading was quantified in ethanol (70%, *v*/*v*) via UV–Vis spectrophotometry with detection at 425 nm. The encapsulation efficiency (*EE*%) and loading capacity (*LC*%) were calculated using Equations (2) and (3):

(1) $$Yield\;(\%)=\frac{m_{final}}{{Cur}_{added}+{carrier}_{added}}\times 100$$

(2) $$EE\left(\%\right)=\frac{{Cur}_{added}-{Cur}_{unbound}}{{Cur}_{added}}\times 100$$

(3) $$LC\left(\%\right)=\frac{{Cur}_{added}-{Cur}_{unbound}}{finalmass}\times 100$$

Zeta potential (0.1%, *w*/*v*, aqueous suspensions) was evaluated via dynamic light scattering (DLS) using a Malvern Instrument Zetasizer Nano ZSP (Malvern, UK). The measurements were taken at room temperature (25 °C) using a folded capillary cell at a constant detection angle of 173°. The equipment was equipped with a 10 mW He-Ne laser with an emission wavelength of 633 nm.

Particle size was determined via laser diffraction using a Malvern Panalytical Mastersizer 3000 (Malvern, UK). Cellulose delivery systems (refractive index of 1.468 and absorption index of 0.01) were analyzed in distilled water (refractive index of 1.33) at 3500 rpm in the obscuration range of 5–15%, using the Mie theoretical model and the size distribution by volume.

Morphology was evaluated on a Pro Scanning Electron Microscope (ThermoScientific, Waltham, MA, USA), where observations were performed in high vacuum with an acceleration voltage of 5–10 kV. Prior to analysis, the samples were placed in observation stubs covered with double-sided adhesive carbon tape (NEM tape, Nisshin, Japan) and coated with Au/Pd (target SC510-314B from ANAME, S.L., Madrid, Spain) using a Sputter Coater (Polaron, Bad Schwalbach, Germany). The images presented are representative images of the morphology of the samples.

### 3.4. Cell Lines and Culture Conditions

Human colon carcinoma Caco-2 and mucus secreting HT29-MTX cells were obtained from the European Collection of Authenticated Cell Cultures (ECACC, Salisbury, UK). The cells were cultured at 37 °C in a humidified atmosphere consisting of 95% air and 5% CO_2_. Dulbecco’s Modified Eagle’s Medium (DMEM) with 4.5 g/L glucose, L-glutamine and no pyruvate (ThermoScientific, Waltham, MA, USA), supplemented with 10% Fetal Bovine Serum (ThermoScientific, Waltham, MA, USA), Penicillin-Streptomycin-Fungizone (1%, *v*/*v*) (ThermoScientific, Waltham, MA, USA) and Non-Essential Amino Acids Solution (1%, *v*/*v*) (MEM NEAA, ThermoScientific, Waltham, MA, USA), in the case of Caco-2 cells. Caco-2 cells were used between passages 28 and 32, and HT29-MTX were used between passages 17 and 21.

### 3.5. Antioxidant Potential

#### 3.5.1. 2,2-Diphenyl-1-Picrylhydrazyl-Free-Radical (DPPH) Assay

The 2,2-Diphenyl-1-picrylhydrazyl (DPPH)-free-radical scavenging activity was measured according to the method described by Schaich et al. [31], with modifications for a 96-well microplate scale. The stock solution (600 μM) was prepared in methanol and stored in the dark at −20 °C. A daily DPPH solution was prepared by dilution with methanol to achieve an absorbance of 0.600 ± 0.100 at 515 nm. For the assay, 25 μL of sample (biocompatible concentrations of 0.12 g/L for the delivery system and 0.005 g/L (15 µM) for curcumin, determined in our previous work [21]), Trolox (7.5–240 μM), ascorbic acid (0.05 g/L, 280 μM) or solvent were added to 175 μL DPPH daily solution. The mixture was incubated for 30 min at 25 °C and the absorbance was measured at 515 nm with a Synergy H1 plate reader ( Agilent, Santa Clara, CA, USA). The scavenging activity was expressed as percentage (%) reduction in absorbance related to the control. Regression equations between DPPH scavenging and Trolox concentration were calculated, and the results expressed as µmol TE (Trolox equivalent) per gram of sample. The DPPH scavenging effect percentage was determined according to Equation (4):

(4) $$DPPH\;scavenging\;effect\left(\%\right)=\left[\frac{A_{control}-A_{sample}}{A_{control}}\right]\times 100$$

where *A_control_* and *A_sample_* are the absorbances at 515 nm of control and sample, respectively.

#### 3.5.2. Oxygen Radical Absorbance Capacity (ORAC) Assay

The oxygen radical absorbance capacity (ORAC) assay was performed according to Coscueta et al. [71] with small adaptations. The reaction was carried out in phosphate buffer (75 mM, pH 7.4) at 37 °C, using fluorescein as a fluorescent probe and 2,2′-azobis(2-methylpropionamidine) dihydrochloride (APPH) as a free radical generator. Briefly, in black 96-well microplates, samples at the desired biocompatible concentrations (0.12 g/L for the delivery system, 0.005 g/L (15 µM) for curcumin and 0.09 for CNC-CTAB, determined in our previous work [21]), Trolox standards (10–80 μM) and ascorbic acid as antioxidant control (0.05 g/L, 280 μM) were added to fluorescein (70 nM). The plate was then pre-incubated for 10 min at 37 °C during 10 min, following which APPH solution (18 mM) was added to the wells and fluorescence (excitation: 485 nm; emission: 528 nm) was read every minute for 80 min using a microplate reader (Synergy H1, Biotek, Santa Clara, CA, USA). Results were determined by the difference of the areas under fluorescein decay curves of samples and blank. A calibration curve using Trolox was obtained, and the results were expressed as μmol of Trolox equivalent per gram of sample.

#### 3.5.3. Production of Reactive Oxygen Species

Caco-2 cells were grown to ca. 80% confluence, detached using TrypLE Express (ThermoScientific, Waltham, MA, USA) and seeded at a density of 1 × 10^4^ cells/well into 96-well black polystyrene microplates. After 24 h, the culture media was carefully replaced with phenol red-free media supplemented with samples at biocompatible concentrations (0.12 g/L for the delivery system, 0.005 g/L (15 µM) for curcumin, and 0.09 g/L for CNC-CTAB, determined in our previous work [21]), with and without an oxidative stimulus (H_2_O_2_ at 100 µM, Supplementary Information, Figure S1), and the plate was incubated for an additional 24 h. Trolox (15 µM) and ascorbic acid (500 µM) were used as positive controls, while plain phenol-free media with or without H_2_O_2_ was used as the basal activity control. After incubation, 2′7′-dichlorofluorescein diacetate (DCFDA, Sigma-Aldrich, St. Louis, MO, USA) probe was added (25 µM), and the plate was incubated again. Fluorescence intensity measurements (Excitation: 485 nm; Emission: 520 nm) were taken in a BioTek Synergy H1 Microplate Reader (Winooski, VT, USA) at 1 h intervals for 4 to 6 h or until the fluorescence signal reached saturation.

### 3.6. Immunomodulation

The monolayer immunomodulatory assays in Caco-2 were performed as previously described by Costa et al. [38]. Briefly, Caco-2 cells were seeded at a density of 2.5 × 10^5^ cells/well in a 24-well microplate and incubated for 24 h at 37 °C in a 5% CO_2_ atmosphere. Following this, the culture media was carefully replaced with media supplemented with samples at biocompatible concentrations (0.12 g/L for the delivery system, 0.005 g/L (15 µM) for curcumin, and 0.09 for CNC-CTAB, determined in our previous work [21]), with and without an inflammatory stimulus, and the plate was incubated for an additional 24 h. Interleukin-1β (Invitrogen, Waltham, MA, USA) was used as an inflammatory stimulus at a concentration of 0.01 µg/mL, and betamethasone (40 µM) was used as positive anti-inflammatory control. Plain media with and without IL-1β was employed as the basal activity control. At the end of the assay, supernatants were collected, centrifuged to remove debris, and stored at −80 °C for further analysis. The detection of interleukins 6 (IL-6) and 8 (IL-8) and Tumor Necrosis Factor alpha (TNF-α) was carried out using enzyme-linked immunosorbent assay (ELISA) kits. Specifically, the Human IL-6 Elisa Kit High Sensitivity (Abcam, Cambridge, UK), the Legend Max Human Elisa Kit IL-8 and the Legend Max Human Elisa Kit TNF-α (BioLegend, San Diego, CA, USA) were used, according to the manufacturers’ instructions. Protein was extracted from the cells and quantified through Pierce™ BCA Protein Assay Kit (ThermoFisher, UK) and used to normalize the results obtained from ELISA. Cytokine values were expressed in pg/mL of sample. To minimize variability associated with proteomic-based assays, results were expressed as fold change in production relative to the cytokine levels in the basal-stimulated control.

### 3.7. Antimicrobial Activity

The antimicrobial activity of CNC-CTAB-encapsulated curcumin, as well as free curcumin and carrier, was evaluated against *Escherichia coli* ATCC 25922, *Salmonella enteritidis* ATCC 13076, *Listeria innocua* ATCC 33091 and *Campylobacter jejuni* ATCC 33560, using an incorporation method based upon the Clinical and Laboratory Standards Institute guidelines—CLSI-M07-A9 standard. To perform the assay, Mueller–Hinton agar was prepared with final intestinal biocompatible concentrations of 0.12 g/L for the delivery system, 0.005 g/L (15 µM) for free curcumin and 0.09 for free CNC-CTAB, as determined in our previous work against intestinal Caco-2 cells [21]. The agar was inoculated with the selected microorganisms at ca. 10^4^ CFU/drop. The plates were then incubated at 37 °C for 48 h, in an atmosphere of 5% O_2_ in the case of *Campylobacter jejuni*, after which microbial growth inhibition was visually assessed by comparing the plates against a dark matte surface. The presence of inhibition zones indicated antimicrobial activity against the tested microorganisms.

### 3.8. Release Profile under Simulated Digestion

CNC-CTAB encapsulating curcumin, as well as free curcumin and carrier, were submitted to in vitro GU digestion according to the standardized European protocol developed by INFOGEST for static in vitro simulation of GI food digestion [57,58]. Initially, water suspensions of 4.8 g/L for the delivery systems, 0.2 g/L for curcumin and 3.6 g/L for CNC-CTAB were prepared. These suspensions were mixed with simulated gastric fluid (SGF) electrolyte solution to achieve a final ratio of 1:1 (*v*/*v*). The initial concentrations were selected to achieve biocompatible concentrations at the end of the digestion with a 1:10 dilution. For the gastric phase, the pH was adjusted to 3.0 with HCl (6 M), and CaCl_2_(H_2_O)_2_ was added to reach a final concentration of 0.15 mM in SGF. Pepsin (Sigma-Aldrich, St. Louis, MO, USA) was added to achieve an activity of 2000 U/mL in the final digestion mix. Then, the digesta solution was incubated for 120 min at 120 rpm and 37 °C. Afterwards, to simulate the intestine phase, simulated intestinal fluid (SIF) electrolyte solution was added to achieve a final ratio of 1:1 (*v*/*v*). The pH of the solution was adjusted to 7.0 using NaOH (6 M). Pancreatin (trypsin activity of 100 U/mL in the final mix) (Sigma-Aldrich, St. Louis, MO, USA) and bile salts (10 mM) (Oxoid ™, Basingstoke, Hampshire, UK) were added to the digesta solution, and CaCl_2_(H_2_O)_2_ was incorporated to a final concentration of 0.6 mM in SGF. The mixture was incubated for 120 min at 120 rpm and 37 °C. Aliquots of 300 µL were withdrawn every hour and added to 700 µL of ethanol. The samples were centrifuged, and curcumin was quantified as described above. After completing the digestion (i.e., 4 h), enzymatic activity was inactivated by heating the samples to 80 °C for 10 min.

### 3.9. In Vitro Intestinal Permeability

An in vitro Caco-2/HT29-MTX co-culture model was used to determine the intestinal permeability of the free and encapsulated curcumin after digestion [72]. Co-culture model was established following the methods previously described by Antunes et al. [73]. Briefly, Caco-2/HT29-MTX co-cultures were seeded onto the apical chamber of 12-well transwell plates (Corning, New York, NY, USA) in a ratio of 90:10, respectively, to reach a monolayer with a final cell density of 1 × 10^5^ cells/cm^2^ in each insert. The apical and basolateral compartments were supplemented with 0.5 and 1.5 mL of media, respectively. The media was replaced three times a week throughout a period of 21 days, and the transepithelial electrical resistance (TEER) was measured to validate the barrier integrity using a Millicell^®^ ERS-2 voltohmmeter (Merck Millipore, Billerica, MA, USA). TEER values, expressed in Ω∙cm^2^, were calculated by subtracting the TEER of the cell-free transwell plates from the TEER of the cell-cultured transwell inserts and multiplying the result by the surface area of the well. Only the monolayers with TEER values superior to 250 Ω·cm^2^ were selected for the subsequent permeability experiments. On the day of the permeability experiment, the culture medium was aspirated from the Caco-2/HT29-MTX co-cultures, the cells were washed twice with 200 μL of Hanks’ Balanced Salt Solution (HBSS) and incubated for 15 min (at 37 °C and 5% CO_2_) with 200 μL HBSS solution (transporter buffer). For the permeability experiment, 0.5 mL of 1:10 dilutions of digested encapsulated and free curcumin, as well as the free carrier, were added to the apical side of the transwell insert. In order to maintain well conditions, 1.5 mL of HBSS was added to the basolateral side. The permeability was determined at 0, 15, 30, 90, 150 and 210 min, by collecting 200 μL aliquots from the basolateral compartment for curcumin quantification. At each time point, the basolateral compartment was replenished with 200 μL of fresh HBSS. Curcumin quantification was performed as described above, after the addition of ethanol (70%, *v*/*v*) and sonication (10 min, 80% amplitude, 500 W, 20 kHz) to ensure particle burst and complete solubilization of the biomolecule. The apparent permeability coefficient (*P_app_*) was determined using Equation (5):

(5) $$P_{app\;(\mathrm{cm}/s)}=\frac{dQ}{dt\times A\times C_{0}}$$

where *dQ* is the total amount of permeated curcumin (μg), *A* is the diffusion area (1.12 cm^2^), *C*_0_ is the initial concentration in the apical compartment (μg/mL), and *dt* is the time of experiment. The coefficient *dQ*/*dt* represents the flux of the test compound across the cell layers [74].

### 3.10. Statistical Analysis

All analyses were performed in triplicate and the results are reported as the mean values and standard deviations. As long as the data followed a normal distribution by the Shapiro–Wilk test, one-way analysis of variance (ANOVA) followed by post hoc Tukey’s test (*p* < 0.05) was conducted to determine the significant differences between mean values using the STATISTICA 10.0 Software Program (TIBCO Software Inc., Palo Alto, CA, USA).

## 4. Conclusions

In this work, the biological potential (antioxidant, anti-inflammatory and antimicrobial activities) of spray-dried CNC-CTAB delivery system encapsulating curcumin was assessed in vitro, following our previous work that demonstrated the potential of said delivery system in the encapsulation, sustained release and cytotoxicity mitigation of curcumin. Furthermore, the effects of curcumin encapsulation on the bioavailability of curcumin were accessed through intestinal permeability studies. Investigations using Caco-2 cells revealed that encapsulated curcumin was able to effectively reduce the production of reactive oxygen species (ROS) induced by hydrogen peroxide, demonstrating its potential to counteract oxidative stress. The encapsulated system exhibited superior ROS-reducing capacity compared to free curcumin at safe concentrations for intestinal cells. Regarding anti-inflammatory response, curcumin’s effectiveness decreased after encapsulation, which may be attributed to the pro-inflammatory effect of the carrier material. Nevertheless, the encapsulated systems were able to reduce cytokine production to levels comparable to the anti-inflammatory agent betamethasone, indicating anti-inflammatory potential. The antimicrobial properties of the CNC-CTAB delivery system against Campylobacter jejuni suggests its potential as a beneficial approach for targeting this pathogen and mitigating associated inflammation in the gastrointestinal tract. The system’s ability to protect curcumin from degradation and facilitate its interaction with the intestinal epithelium through mucoadhesive properties opens up possibilities for delivering higher amounts of curcumin and enhancing its biological functions towards intestinal cells and pathogens. Given the role of inflammation, redox-mediated dysregulation and the presence of *Campylobacter jejuni* in inflammatory intestinal diseases, the findings of this study highlight the potential of CNC-CTAB as a promising carrier for improving the biological functions of curcumin, supporting its further investigation as potential beneficial agent against intestinal inflammatory disorders. The findings of this study are significant, as to the best of our knowledge, this is the first study investigating the biological potential, release profile under gastrointestinal conditions and intestinal permeability of CNC-CTAB delivery systems encapsulating curcumin.

## Figures and Tables

**Table 1:** Yield, encapsulation efficiency (EE), loading capacity (LC), zeta potential (ZP) and size of CNC-CTAB particles encapsulating curcumin.

Delivery System	Yield (%)	EE (%)	LC (%)	Zeta Potential (mV)	Particle Size	
					Dv 50 (µm)	D 4:3 (µm)
CNC-CTAB_Curcumin	81.97	82.61 ± 0.45	28.63 ± 0.31	−17.73 ± 0.30	7.33	9.34

Legend: CNC—cellulose nanocrystals; CTAB—modified with cetyltrimethylammonium bromide; Dv 50—median volumetric diameter; D 4:3—volume-weighted mean diameter.

**Table 2:** Antimicrobial activity of CNC-CTAB encapsulating curcumin (0.12 g/L), curcumin (15 µM) and free CNC-CTAB (0.09 g/L) against four GI tract reference microorganisms using the incorporation method.

Sample	*E. coli*	*S. enteritidis*	*L. innocua*	*C. jejuni*
CNC-CTAB_Cur	−	−	−	+
Curcumin	−	−	−	−
CNC-CTAB	−	−	−	+

Legend: (−) No antimicrobial effect (microorganism growth detected); (+) antimicrobial effect (inhibition of the microbial growth); tested microorganisms: *Escherichia coli*, *Salmonella enteritidis*, *Listeria innocua* and *Campylobacter jejuni*.

## Data Availability

The datasets used and/or analyzed during the current study are available from the corresponding author on reasonable request.

## Supplementary Materials

The following supporting information can be downloaded at: https://www.mdpi.com/article/10.3390/ph16121737/s1. Figure S1. Impact of H_2_O_2_ at various concentrations (10 µM–1 M) upon Caco-2 cells metabolic activity; Figure S2. Impact of various dilutions of the digested CNC-CTAB system encapsulating curcumin, free curcumin and free CNC-CTAB upon Caco-2 and HT29-HTX cells metabolic activity.
