# Supplementary material for: Biological Potential and Bioaccessibility of Encapsulated Curcumin into Cetyltrimethylammonium Bromide Modified Cellulose Nanocrystals
Source: Pharmaceuticals (Basel). 2023 Dec 17;16(12):1737. doi: 10.3390/ph16121737 (PMC10747507; doi:10.3390/ph16121737)
Supplement: Supplementary file 1 [file pharmaceuticals-16-01737-s001.zip › pharmaceuticals-2772581-supplementary.pdf]

Article

Biological Potential and Bioacessibility of Encapsulated Curcumin into CTAB Modified Cellulose Nanocrystals

Francisca Casanova *, Carla F. Pereira, Alessandra B. Ribeiro, Pedro Castro, Ricardo Freixo, Eva Martins, Diana Valente, João Fernandes, Manuela E. Pintado and Óscar L. Ramos *

CBQF—Centro de Biotecnologia e Química Fina-Laboratório Associado, Escola Superior de Biotecnologia, Universidade Católica Portuguesa, Rua Diogo Botelho 1327, 4169-005 Porto, Portugal

* Correspondence: fcbastos@ucp.pt (F.C.); oramos@ucp.pt (Ó.L.R.)

Supplementary Information

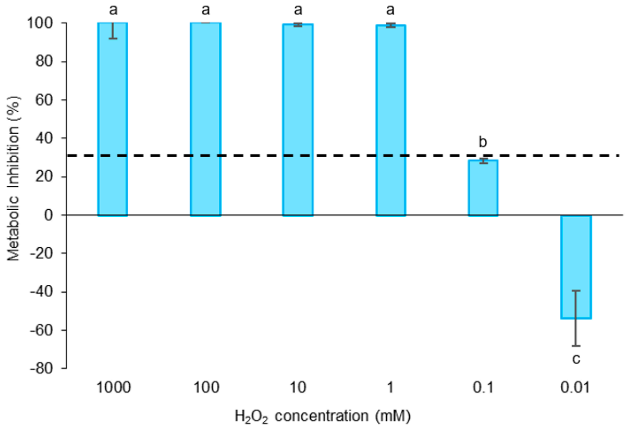

Figure S1. Impact of H_2O_2 at various concentrations (10 µM -1 M) upon Caco-2 cells metabolic activity. The dotted line represents the 30% cytotoxicity limit as defined by the ISO 10993-5:2. Different letters represent the statistically significant ($p < 0.05$) differences found between samples.

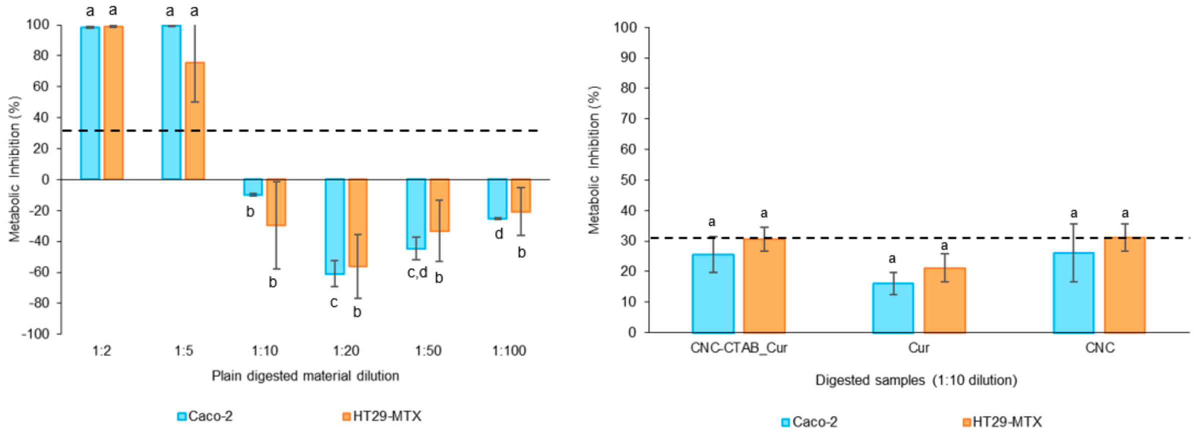

Figure S2. Impact of various dilutions of the digested CNC-CTAB system encapsulating curcumin, free curcumin and free CNC-CTAB upon Caco-2 and HT29-HTX cells metabolic activity. The dotted line represents the 30% cytotoxicity limit as defined by the ISO 10993-5:2. Different letters represent the statistically significant ($p < 0.05$) differences found between the samples tested for each cell line assayed.
